# Evaluation of the Performance of a YOLOv10-Based Deep Learning Model for Tooth Detection and Numbering on Panoramic Radiographs of Patients in the Mixed Dentition Period

**DOI:** 10.3390/diagnostics15040405

**Published:** 2025-02-07

**Authors:** Ramazan Berkay Peker, Celal Oguz Kurtoglu

**Affiliations:** 1 Department of Dentomaxillofacial Radiology, Faculty of Dentistry, Trakya University, Edirne 22030, Turkey; 2 Department of Computer Engineering, Faculty of Engineering, Cukurova University, Adana 01330, Turkey; celaloguz.kurtoglu@gmail.com

**Keywords:** artificial intelligence, YOLO, tooth classification, panoramic radiography, pediatric dentistry

## Abstract

**Objectives:** This study evaluated the performance of a YOLOv10-based deep learning model in detecting and numbering teeth in the panoramic radiographs of pediatric patients in the mixed dentition period. **Methods:** Panoramic radiographic images from 200 pediatric patients in the mixed dentition period, each with at least 10 primary teeth and underlying permanent tooth germs, were included in the study. A total of 8153 teeth in these panoramic radiographs were manually labeled. The dataset was divided for the development of a YOLOv10-based artificial intelligence model, with 70% used for training, 15% for testing, and 15% for validation. **Results:** The precision, recall, mAP50, mAP50-95, and F1 score of the model for tooth detection and numbering were found to be 0.90, 0.94, 0.968, 0.696, and 0.919, respectively. **Conclusions:** YOLOv10-based deep learning models can be used to accurately detect and number primary and permanent teeth in the panoramic radiographs of pediatric patients in the mixed dentition period, which can support clinicians in their daily practice. Future works may focus on model optimization across varied pediatric cases to enhance clinical applicability.

## 1. Introduction

Panoramic radiographs (PRs), introduced in the 1950s, have become a significant and widely used diagnostic technique in dentistry [1]. One of the primary advantages of PRs is their ability to capture the maxilla, mandible, temporomandibular joints, and associated anatomical structures within a single image [2,3]. However, the superimposition and distortion of anatomical structures due to the two-dimensional nature of PRs can hinder accurate evaluation [4]. Despite these limitations, the advantages of PRs—such as the relatively low radiation dose, the ability to image a broad area within a short clinical timeframe, and procedural simplicity—make them a frequent choice as an initial diagnostic tool in pediatric dentistry [3].

Accurate interpretation of PRs is essential for correct diagnosis and, consequently, appropriate treatment. In the mixed dentition period, particularly in children, identifying and numbering both erupted and unerupted teeth is critical for treatment planning and prioritizing necessary interventions. However, manual numbering of erupted and unerupted teeth is a time-consuming process. Furthermore, the accuracy of this numbering varies with the experience of the examining dentist [5,6].

Artificial intelligence (AI) is a broad term for computer systems designed to perform tasks that typically require human intelligence, such as visual perception and decision-making [3]. The use of AI-based methods to assist dentists in interpreting images enables faster identification and classification of data. These applications also help eliminate errors associated with human factors, such as fatigue and lack of experience [6,7].

In dental research, machine learning (ML), a subset of AI, focuses on developing algorithms that can learn from data to make predictions or decisions without explicit programming and to improve performance as they encounter more data [3]. Deep learning (DL), a subfield of ML, involves techniques that automatically learn from datasets through the use of convolutional neural networks (CNNs), a type of deep learning model (DLM). Rather than following explicit instructions, CNNs learn by creating models based on data analysis [5,8].

You Only Look Once (YOLO), a DL algorithm, can be directly and independently trained on validation data, enabling it to detect multiple objects in an image in real time without the need for a reference dataset. Due to its high detection speed, accuracy, and generalization capability, YOLO is among the most popular CNNs for object segmentation and detection [9,10].

YOLOv10, developed by researchers at Tsinghua University and released in May 2024, is considered a significant advancement in real-time object detection [11]. This new architecture aims to balance accuracy and computational efficiency to address the challenges of object detection. YOLOv10 achieves this through innovative training strategies, architectural modifications, and a combination of model variants. The core concept involves using “consistent dual assignment” during training, which allows the model to learn from rich supervision signals while eliminating the need for computationally expensive non-maximum suppression (NMS) during inference. Extensive evaluations have demonstrated that YOLOv10 surpasses previous YOLO versions and other state-of-the-art ML models in terms of accuracy–efficiency balance [11].

Several studies have employed DLMs for tooth detection and numbering in PRs [6,12,13,14]. However, few studies have focused on the mixed dentition period [5]. To our knowledge, no study has investigated the use of YOLOv10 for tooth segmentation and detection during the mixed dentition period. This study evaluates the effectiveness of YOLOv10 in the automatic detection and numbering of teeth in pediatric patients in the mixed dentition period, thereby contributing to both dentomaxillofacial radiology and pediatric dentistry. The study hypothesizes that YOLOv10 will successfully detect and number both primary and permanent teeth.

## 2. Materials and Methods

The preparation of this manuscript followed the procedures outlined in the Checklist for Artificial Intelligence in Medical Imaging (CLAIM) and the Standards for the Reporting of Diagnostic Accuracy Studies (STARD). This study was approved by the Ethics Committee of Trakya University Faculty of Medicine (Approval number: TÜTF-GOBEAK 2024/450). All the procedures followed were in accordance with the ethical standards of the responsible committee on human experimentation (institutional and national) and the Declaration of Helsinki. Informed consent was waived because the study involved a retrospective analysis of previously collected and de-identified panoramic radiographs.

### 2.1. Overview of the Study Workflow

**Figure 1:**
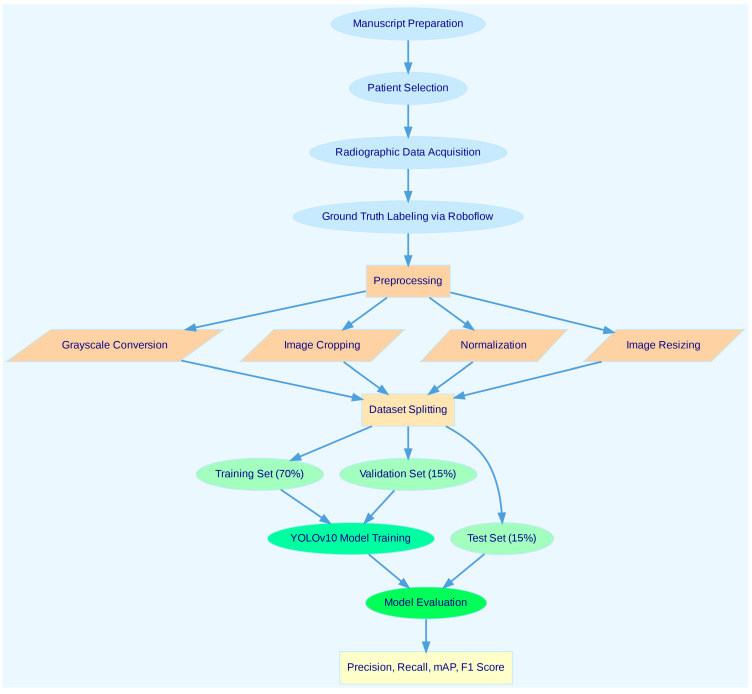
Workflow of the study. The flowchart illustrates the key steps undertaken in this research, including patient selection, data preprocessing, labeling, YOLOv10 model training, validation, and performance evaluation. Each step is detailed to provide a clear and concise overview of the methodology, ensuring reproducibility and aiding in the interpretation of the study results.

To provide a clear and concise representation of the methodology, a flowchart summarizing the study workflow is presented in Figure 1. This flowchart outlines the key steps, including patient selection, data preprocessing, labeling, model training, validation, and performance evaluation. It is intended to improve the readability and reproducibility of the study by visually guiding the reader through the methodological process.

### 2.2. Patient Selection

In this retrospective study, we analyzed the PRs of pediatric patients aged 5 to 13, which were obtained from the archives of the Department of Oral and Maxillofacial Radiology at (Blinded for Review) University Faculty of Dentistry from 2019 to 2024. Patients with PRs containing artifacts (e.g., motion or metal artifacts), those with cystic or tumoral formations in the jaw, developmental abnormalities (e.g., cleft lip or palate), or positioning errors during PR acquisition were excluded from the study. A total of 200 patient images, comprising PRs classified as Grade 1 or Grade 2 according to the UK National Radiological Protection Board criteria, were included in the study [12]. These PRs contained at least 10 primary teeth with underlying permanent tooth germs. The presence of caries, restorative fillings, root canal treatments, tooth rotation, or supernumerary teeth in the included cases was not considered an exclusion criterion.

### 2.3. Radiographic Dataset

The PRs were acquired by a single operator using standardized patient positioning on a PaX-Flex radiography device (Vatech, Seoul, Republic of Korea) with settings of 50–90 kV and 4–10 mA and an exposure time of 10.1 s.

### 2.4. Ground Truth Labeling

**Figure 2:**
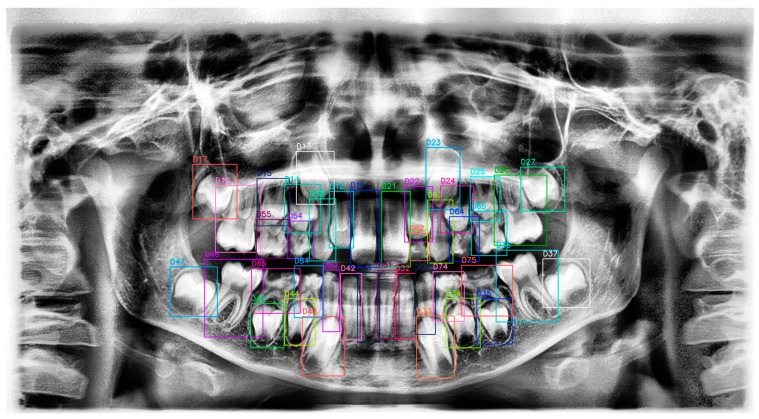
Panoramic radiograph with tooth annotations by R.B.P. for YOLOv10 training. This figure displays a panoramic radiograph where each tooth has been manually annotated with labels and bounding boxes by R.B.P. for the purpose of training the YOLOv10 model. The annotations include distinct colors and identifiers for each tooth, providing a comprehensive dataset for model training and ensuring accurate tooth localization and classification. This manually labeled image serves as ground truth data to enhance the YOLOv10 model’s performance in tooth detection tasks.

The labeling process enables ML or DL models to derive meaningful contexts related to objects in an image. Within these contexts, the classes and regions of the objects are identified. In this study, labeling was carried out by R.B.P., an oral and maxillofacial radiologist with seven years of experience (Figure 2). Two weeks after the initial labeling, all PRs were reevaluated by R.B.P. The labeling process was conducted on the Roboflow website (https://roboflow.com/, accessed on 19 November 2024) using the bounding box method. The software used for dataset preparation and labeling, including Roboflow, offers both free and paid plans depending on the user’s needs. Labels were created in TXT format to be compatible with YOLOv10.

Before labeling, the names, genders, and birth dates of the patients were removed from the PRs to ensure full anonymity. The numbering of primary and permanent teeth in the labeling process was carried out according to the Fédération Dentaire Internationale (FDI) tooth numbering system.

### 2.5. YOLOv10

YOLOv10 is an advanced version of the YOLO series of real-time object detection models designed to enhance both accuracy and efficiency. Building on the strengths of its predecessors, it incorporates innovative features that make it particularly effective for edge applications in which computational resources are limited. Previous YOLO models often involved steps like NMS, which could introduce delays and make seamless integration challenging. YOLOv10 addresses these issues by offering a training strategy that focuses on efficiency and accuracy and does not require NMS.

By using consistent dual-assignment methods, YOLOv10 enables training without NMS, achieving both competitive performance and low latency. Additionally, it proposes a comprehensive design strategy that optimizes the various components of YOLO for both efficiency and accuracy. This reduces computational load and enhances performance. YOLOv10 is offered in multiple versions to meet users’ different computational requirements and performance needs. These versions vary in their number of parameters, allowing users to select the model suited to their hardware and specific application scenarios.

In this study, the open-source and freely available Extra Large (x) version of YOLOv10 was used to achieve the highest possible accuracy for non-commercial purposes.

#### 2.5.1. Analysis of the YOLOv10 Architecture

The YOLOv10 architecture introduces several significant innovations by building on the strengths of previous versions. The main components of the model are as follows:

*Backbone:* The backbone used for feature extraction in YOLOv10 is equipped with an enhanced version of Cross Stage Partial Network (CSPNet). This structure improves gradient flow and reduces computational load.

*Neck:* The neck is designed to aggregate features at different scales and transfer them to the head. It uses Path Aggregation Network (PAN) layers to effectively combine these features.

*One-to-Many Head:* During training, it generates multiple predictions for each object, providing more supervision signals and increasing learning accuracy.

*One-to-One Head:* During the inference stage, it provides a single optimal prediction for each object. This approach eliminates the need for NMS, reducing latency and enhancing efficiency.

#### 2.5.2. Key Features of YOLOv10

*NMS-free training:* YOLOv10 eliminates the need for NMS by using consistent dual-assignment methods. This approach reduces latency during inference and provides faster results.

*Holistic model design:* The model is comprehensively optimized for both efficiency and accuracy. These optimizations include elements such as lightweight classification heads, spatial-channel separated downsampling, and rank-guided block design.

*Enhanced model capabilities:* YOLOv10 integrates large-kernel convolutions and partial self-attention modules. These features enhance performance without significantly increasing computational costs.

### 2.6. Preprocessing

Various image preprocessing techniques were applied to enhance data quality and optimize model performance. The preprocessing tasks described in the manuscript, such as grayscale image conversion, image cropping, normalization, and resizing, were performed using automated scripts within the software environment. These tasks were implemented programmatically to minimize manual intervention and ensure consistency across the dataset. The operator’s role in the preprocessing stage was limited to verifying the quality of the input images and ensuring that they met the inclusion criteria. By automating most of the preprocessing steps, the workflow was designed to save time and reduce human effort compared to fully manual preprocessing methods. This balance between automation and human oversight ensured efficiency and consistency throughout the preprocessing workflow. The detailed implementation of these techniques is described below:

- *Grayscale image loading:* Although panoramic radiographs are typically acquired in grayscale, all images were explicitly converted to a single-channel grayscale format during preprocessing. This step was undertaken to ensure uniformity across the dataset by eliminating potential inconsistencies in image format or metadata, which might arise due to differences in file handling or storage protocols.
- *Image cropping:* Specific regions of the images were cropped to focus solely on important areas. This approach eliminated distracting elements, allowing the model to work with only the necessary data during the training process.
- *Contrast enhancement:* The contrast of the images was increased to make the details more prominent. This process enabled the model to learn important features better in low-contrast images.
- *Normalization:* Pixel values were normalized to the (0, 1) range. Normalization helped achieve more consistent results by reducing the impact of brightness and contrast variations across images.
- *Image resizing:* Images were resized to 640 × 640 pixels. This standardized sizing ensured consistency in the model’s inputs and eliminated data mismatches during the training process.

### 2.7. Dataset Splitting

The dataset, which consisted of 200 images (with a total of 8153 labels) used to train and evaluate the YOLOv10 DLM, was split in the following manner:

- *Training set:* 70% of the total dataset, comprising 140 images, was used to train the YOLOv10 DLM. This portion was allocated to allow the model to learn various object features and improve its overall performance.
- *Test set:* 15% of the dataset, consisting of 30 images, was set aside for independent testing of the model. This portion was used to assess the model’s performance on real-world data and evaluate its accuracy.
- *Validation set:* The validation set, comprising 15% of the dataset, was used to fine-tune the model’s hyperparameters and monitor performance. During training, validation metrics such as precision, recall, and loss values were evaluated at each epoch to assess the model’s ability to generalize to unseen data. Early stopping was implemented to halt training if no improvement was observed in validation loss for 10 consecutive epochs, thereby minimizing the risk of overfitting. The validation set was independent of both the training and test datasets, ensuring unbiased evaluation of the model.

Data splitting ensured a balanced and effective evaluation of YOLOv10 during the training, testing, and validation processes, allowing for accurate measurement and optimization of the model’s overall performance.

### 2.8. YOLOv10 Model Training

YOLOv10 stands out as one of the most advanced and powerful object detection models. In this study, YOLOv10 was trained on an Nvidia RTX 4070 Ti GPU, and the parameters used during the training process were examined.

#### 2.8.1. Training Process and Parameters

- *model:* Set to ‘None’, meaning that no specific model file was designated. In this case, a default model configuration file or a pretrained model was not used.
- *data:* Specified as ‘None’, indicating that no configuration file for dataset to be used in training was defined.

#### 2.8.2. Training Duration and Early Stopping

- *epochs:* Training was set to a total of 200 epochs, meaning the model was trained on the 70% training subset 200 times. This iterative process allowed the model to optimize its parameters by repeatedly learning from the training data.
- *time:* Set to ‘None’, indicating that no maximum training time was restricted. Training was completed based on the specified number of epochs.
- *patience:* Early stopping was applied if no improvement was observed for 10 consecutive epochs. This aimed to prevent the model from overfitting.

#### 2.8.3. Batch and Image Size

- *batch:* The batch size used during training was set to 16. This represents the number of samples processed by the model at each step, which affects processing speed.
- *imgsz:* The image size was set to 640 pixels, meaning that all training images would be resized to this dimension.

#### 2.8.4. Saving and Caching

- *save:* Set to ‘True’, indicating that the model would be saved during training.
- *save period:* Set to ‘−1’, meaning the model would not be saved at the end of each epoch.
- *cache:* Set to ‘False’, indicating that the dataset would not be cached in memory.

#### 2.8.5. Optimization and Other Parameters

- *optimizer:* Set to ‘Auto’, meaning that the most suitable optimization method was automatically selected based on model performance.
- *cos_lr:* Set to ‘False’, meaning that a cosine curve scheduler was not applied to the learning rate.
- *amp:* Set to ‘True’, indicating that Automatic Mixed Precision training was enabled to enhance computational efficiency.
- *fraction:* The entire dataset (1.0) was used for training.
- *freeze:* Set to ‘None’, meaning that no layers of the model were frozen, and all layers were trained.

Parameters such as training duration, batch size, and image size were selected to optimize the model’s performance and accuracy.

YOLOv10 was trained on a dataset with 52 classes. The training process lasted for a total of 200 epochs to maximize model performance. An early stopping strategy was applied to prevent overfitting and achieve optimal results. The early stopping criterion was set to terminate training if no improvement was observed within the last 10 epochs.

Throughout the training process, the model was carefully monitored, and the best results were achieved at epoch 71. After this epoch, no further improvements in model performance were observed, leading to an automatic termination of the training process. The model with the best performance, recorded at epoch 71, was saved as “best.pt” with the corresponding weights. This approach optimized model performance while enhancing the efficiency of the training process and minimizing the risk of overfitting. The final results demonstrate that YOLOv10 performed effectively across a wide range of classes and that the early stopping strategy contributed significantly to the training process.

### 2.9. Metrics of Model Performance

The performance of the YOLOv10 for tooth detection and numbering was evaluated using the following metrics: precision, recall, mean Average Precision at Intersection over Union (IoU) threshold of 0.5 (mAP50), mean Average Precision across IoU thresholds from 0.5 to 0.95 (mAP50-95), and F1 score. Precision measures the proportion of correctly identified teeth among all detections, while recall indicates the proportion of true positives identified. mAP50 and mAP50-95 provide comprehensive measures of detection accuracy across varying thresholds, and the F1 score represents the harmonic mean of precision and recall, offering a balanced evaluation of model performance.

Various metrics were used to comprehensively evaluate model performance. During the training process, train/box_loss, train/cls_loss, and train/dfl_loss losses were analyzed. These losses were used to measure the accuracy of the model’s box predictions, class predictions, and detailed feature predictions, respectively.

The model’s prediction accuracy and overall performance were evaluated using metrics such as metrics/precision, metrics/recall, metrics/mAP50, and metrics/mAP50-95. Precision and recall assess the accuracy and coverage of the model’s positive predictions, while mAP50 and mAP50-95 measure its overall success rate across various threshold values.

During the validation phase, val/box_loss, val/cls_loss, and val/dfl_loss values were examined. These validation losses were used to assess the model’s performance on real-world data outside of the training set.

To optimize the training process, learning rates lr/pg0, lr/pg1, and lr/pg2 were set. By adjusting learning rates for different parameter groups, the aim was to improve the speed and efficiency of the model’s learning process.

To analyze the model’s performance more comprehensively, the F1 score and confusion matrix were utilized. The F1 score provided the harmonic mean of precision and recall, while the confusion matrix helped visualize the model’s correct and incorrect predictions in detail.

## 3. Results

**Figure 3:**
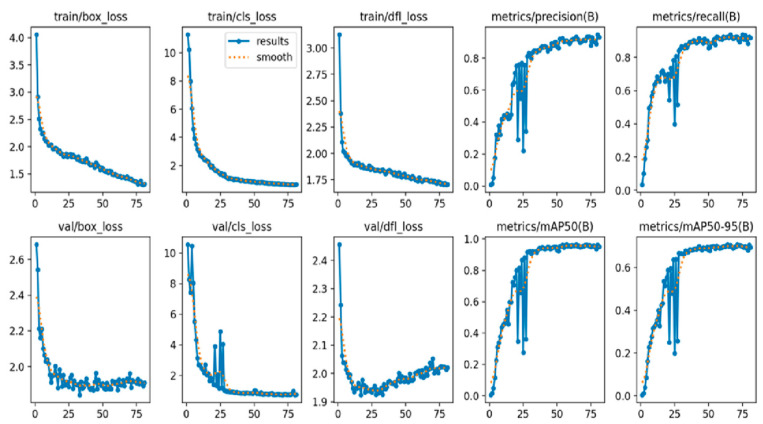
Training and validation metrics for YOLOv10. This figure displays the training and validation loss metrics, including box loss, classification loss, and distribution focal loss, alongside performance metrics of precision, recall, mAP50, and mAP50-95 across 75 epochs. The top row represents training metrics, while the bottom row shows validation metrics. The solid blue line indicates raw results, and the dotted orange line represents the smoothed trend, helping to visualize the model’s performance improvement over the training process.

**Figure 4:**
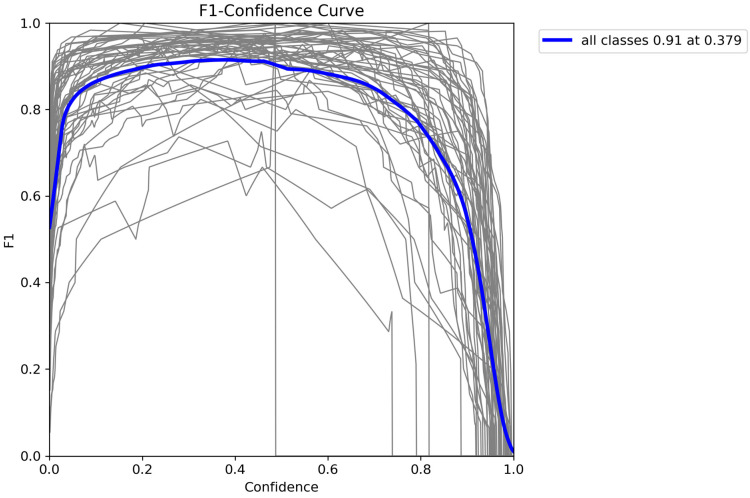
F1-confidence curve for YOLOv10. This figure illustrates the F1-Confidence curve for the YOLOv10 across all classes. The blue line represents the average F1 score across different confidence thresholds, peaking at an F1 score of 0.91 at a confidence level of 0.379. The gray lines depict F1 scores for individual classes, showing the model’s performance variance across classes. This curve provides insights into the optimal confidence threshold that maximizes the F1 score, indicating the balance between precision and recall for effective tooth detection and classification.

**Figure 5:**
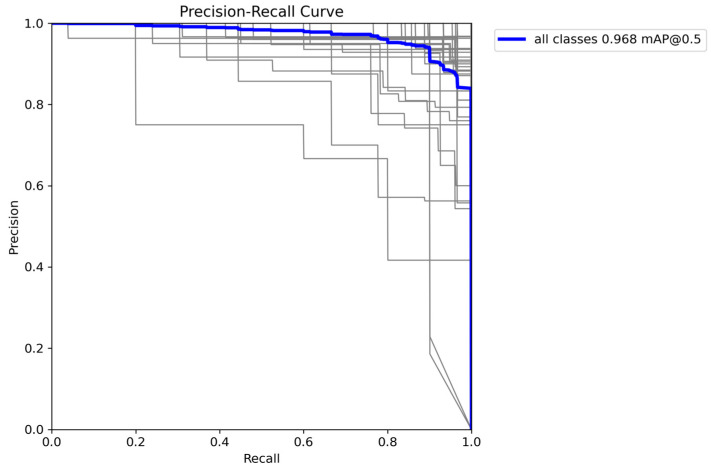
Precision-recall curve for YOLOv10. This figure shows the precision-recall curve for the YOLOv10 across all classes, with an overall mAP (mean Average Precision) of 0.968 at an IoU (Intersection over Union) threshold of 0.5. The blue line represents the average performance across all classes, while the gray lines illustrate the precision-recall relationship for individual classes. This curve highlights the model’s ability to maintain high precision as recall increases, indicating strong detection accuracy for the tooth classification task.

**Figure 6:**
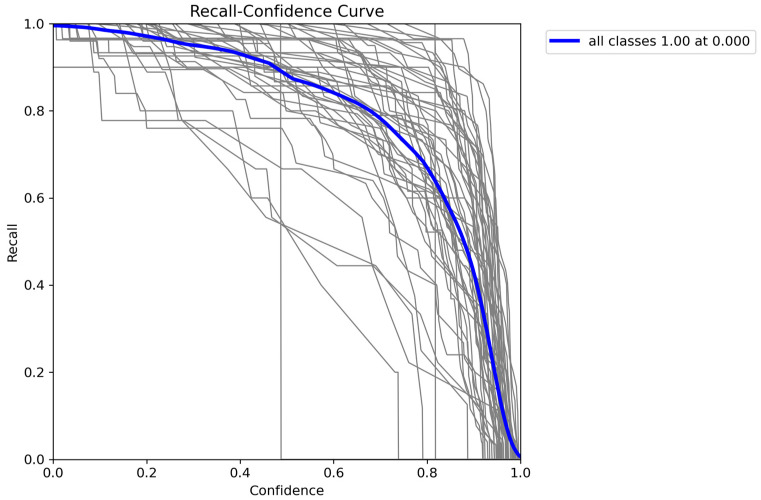
Recall-confidence curve for YOLOv10. This figure shows the recall-confidence curve for the YOLOv10 across all classes. The blue line represents the average recall at different confidence thresholds, achieving a maximum recall of 1.00 at a confidence level of 0.000. The gray lines depict the recall for individual classes, illustrating the model’s behavior as confidence levels increase. This curve is useful for analyzing how changes in confidence thresholds impact the model’s recall, providing insights into its detection capabilities and threshold selection for optimal performance.

**Figure 7:**
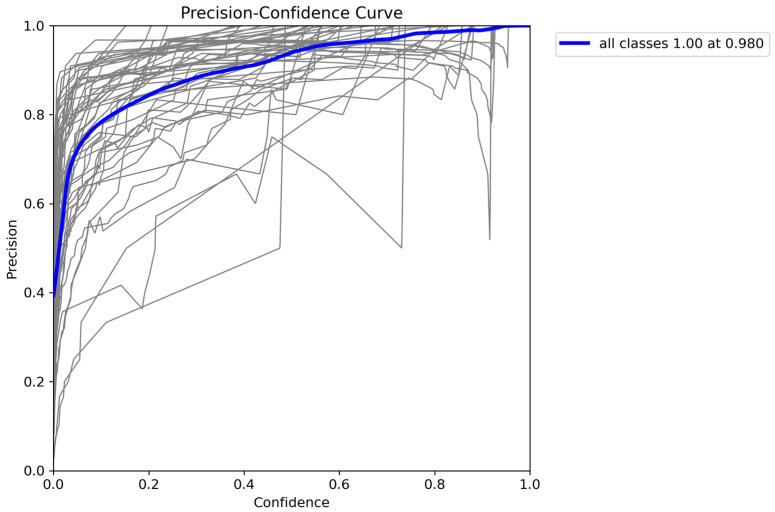
Precision-Confidence Curve for YOLOv10 Model. This figure illustrates the precision-confidence curve for the YOLOv10 across all classes. The blue line represents the average precision at different confidence thresholds, reaching a maximum precision of 1.00 at a confidence level of 0.980. The gray lines show precision values for individual classes, highlighting variations in performance across different confidence levels. This curve helps in understanding how adjusting the confidence threshold impacts precision, providing insights into optimizing the model’s performance for tooth detection and classification tasks.

**Figure 8:**
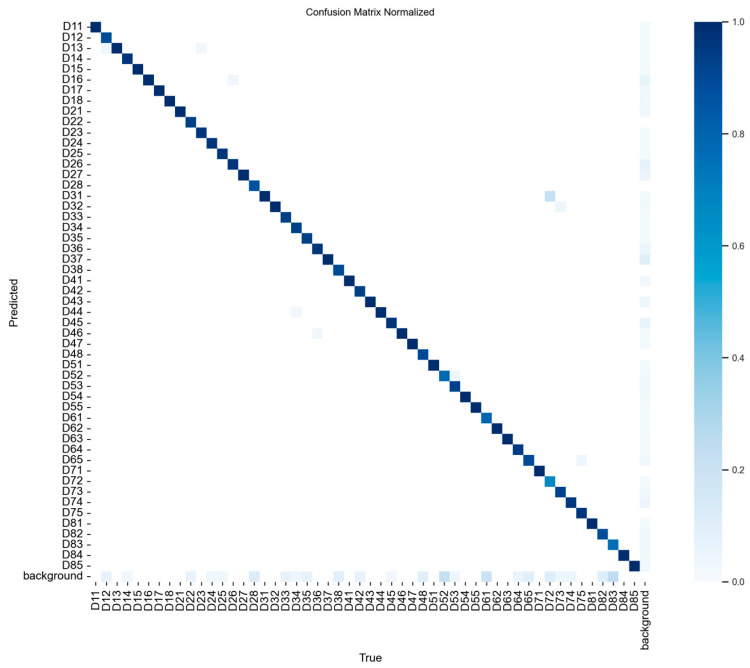
Normalized confusion matrix for tooth detection and classification with YOLOv10. This figure presents the normalized confusion matrix for the YOLOv10’s tooth detection and classification performance across various tooth classes. Each cell represents the model’s prediction accuracy for a specific class, with diagonal cells indicating correct predictions and off-diagonal cells showing misclassifications. The color intensity corresponds to the proportion of predictions, with darker shades indicating higher accuracy. The background class is also included, highlighting the model’s ability to distinguish teeth from non-tooth regions. This matrix provides insight into the model’s classification accuracy and error patterns across different tooth types.

**Figure 9:**
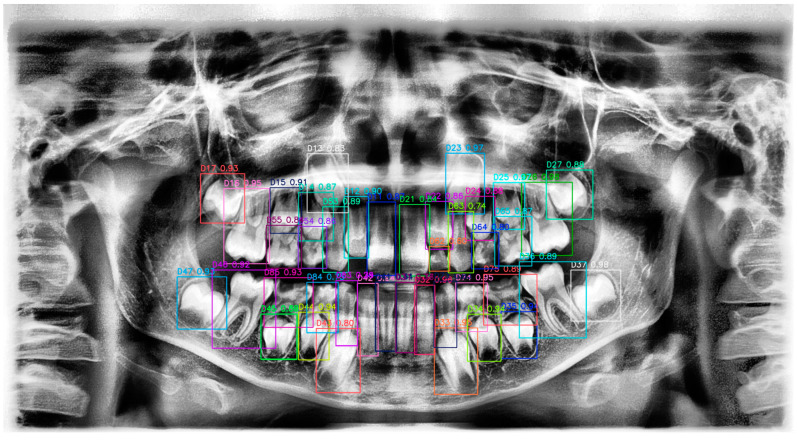
Detected teeth with confidence scores on panoramic radiograph using YOLOv10. This figure shows a panoramic radiograph with bounding boxes indicating the detected tooth regions, labeled by tooth identifiers and confidence scores. Each bounding box represents the YOLOv10’s prediction, with different colors used to distinguish individual teeth. The confidence score for each detection is displayed alongside the corresponding tooth label, indicating the model’s certainty in identifying each tooth. This visualization demonstrates the model’s capability in accurately localizing and classifying teeth within a complex radiographic image.

The weighted average performance metrics of the YOLOv10 DLM for tooth detection and numbering—precision, recall, mAP50, mAP50-95, and F1 score—were 0.90, 0.94, 0.968, 0.696, and 0.919, respectively (Table 1, Figure 3, Figure 4, Figure 5, Figure 6, Figure 7, Figure 8 and Figure 9). These results were obtained exclusively on the test set, meaning a dataset completely independent of the training data was used to evaluate the model’s true performance.

**Figure 10:**
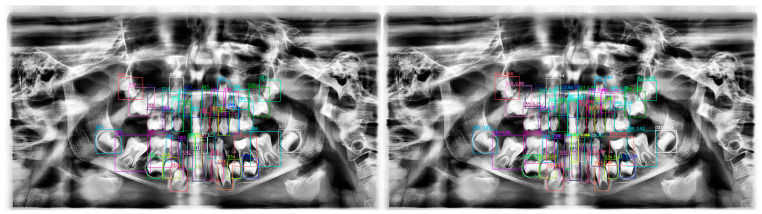
(**Left**) Ground truth labels for the panoramic radiograph. (**Right**) Predictions by the YOLOv10 model. The misclassification of tooth 51 is indicated. Each bounding box represents YOLOv10’s predictions, with different colors distinguishing individual teeth. Confidence scores indicate the model’s certainty for each detection.

The mAP50 for tooth 51 was found to be 0.712, significantly lower than the mAP50 values of other teeth, all of which exceeded 0.9 (Table 1). Further analysis revealed that the YOLOv10 model incorrectly predicted tooth 51 in certain cases, as illustrated in Figure 10. In the left panoramic radiograph in Figure 10, the ground truth labels are displayed, while in the right panoramic radiograph, the model’s predictions are shown. While the model demonstrated strong performance for most teeth, the misclassification of tooth 51 highlights a limitation in its ability to detect this specific tooth accurately.

## 4. Discussion

With the advancement of AI and DL technologies, there is growing interest in integrating these technologies into dentistry. AI and DLMs play a diagnostic and supportive role by enhancing diagnostic accuracy through the analysis of radiographic images. This positively impacts the success of treatments and long-term prognosis [15]. Tooth detection and numbering, which are among the initial steps of accurate diagnosis and treatment, can be a time-consuming and error-prone process when performed manually. Conducting these procedures with the assistance of DLMs not only reduces the clinical workload but also minimizes potential errors.

Numerous studies have utilized various imaging techniques for tooth detection and numbering [5,14,16,17]. Although studies have demonstrated the effectiveness of DLMs [18], they have primarily focused on permanent teeth, with significantly fewer studies targeting mixed dentition.

Ahn et al. compared different DLMs for detecting mesiodens in the PRs of children in the primary or mixed dentition stages and reported that they accurately detected the actual locations of mesiodens in many cases [19]. Similarly, a study examining the detection of supernumerary teeth using CNNs found that they yielded positive results in the early mixed dentition period [20]. Recent studies using earlier versions of YOLOv10, such as YOLOv3 and YOLOv4, have also shown that these DLMs are highly successful in detecting both mesiodens and permanent teeth in PR images obtained from pediatric patients [21,22].

Despite these advancements, research focusing specifically on tooth detection and numbering in pediatric PRs remains limited. Kaya et al. examined YOLOv4’s performance in detecting and numbering primary and permanent teeth in pediatric PRs and reported an mAP of 92.22%, mAR of 94.44%, and weighted F1 score of 0.91, noting that this model could be used as a high-accuracy, fast method for automated tooth detection and numbering [23]. Similarly, Beşer et al. reported precision, recall, F1 score, and mAP-0.5 values all exceeding 0.98 using YOLOv5 for the same purpose [5]. These findings underline the potential of YOLO models in automating tooth detection with high accuracy. In comparison, our study using YOLOv10 achieved a weighted F1 score of 91%, comparable to earlier YOLO models while demonstrating enhanced efficiency and fewer parameters in its architecture. This improvement in efficiency without compromising accuracy makes YOLOv10 particularly well-suited for clinical applications.

In addition to tooth detection and numbering, YOLOv10 can be adapted for other clinical applications such as identifying supernumerary teeth, impacted teeth, or pathologies, making it a versatile tool in pediatric and general dentistry. Furthermore, it can assist in orthodontic treatment planning by monitoring tooth development and positioning. In trauma cases, the model could be used to detect missing or fractured teeth rapidly, aiding emergency dentistry workflows. Such versatility highlights its potential to address a variety of diagnostic and treatment challenges in dental practice.

Other studies have also highlighted the strong performance of different DLM architectures in tooth detection tasks. For instance, Putra et al. used YOLOv4 for automated permanent tooth numbering and reported an F1 score of 93.44%, demonstrating its high reliability and faster detection times compared to manual methods [24]. Similarly, Bilgir et al. employed Faster R-CNN and achieved a sensitivity of 95.59% and an F1 score of 96.06% for tooth numbering, confirming the robustness of DLMs for automated dental diagnostics [25]. Our findings align with these studies, reaffirming that YOLOv10 can perform comparably or better in mixed dentition cases, where the anatomical complexity poses additional challenges.

In our study, we aimed to build on this foundation by evaluating the YOLOv10 model’s performance. The weighted F1 score of 91% achieved in our study confirms the model’s effectiveness in detecting and numbering both primary and permanent teeth in pediatric patients. YOLOv10’s novel architecture, utilizing fewer parameters, enables faster detection and numbering compared to earlier versions, making it a practical choice for clinical applications [11].

However, certain limitations should be acknowledged. The study was conducted at a single center, using PRs obtained exclusively from patients in the Thrace region. While the uniform imaging parameters ensured consistency, the limited population size constrained the sample size, which included only PRs with at least 10 primary teeth and underlying permanent tooth germs to ensure accuracy. This selective approach, while enhancing data reliability, reduced the dataset’s diversity. Furthermore, the emphasis on the F1 score over accuracy was necessary to address the imbalance in the dataset, ensuring a more robust evaluation.

Using data from multiple imaging machines could address these limitations and significantly enhance the model’s generalizability. Different imaging devices often vary in parameters such as resolution, contrast, and exposure settings, which could initially introduce variability in the model’s performance. However, training the model on datasets obtained from multiple machines would expose it to a broader range of imaging conditions and artifacts, improving its robustness and adaptability. Such an approach would make the model more suitable for deployment in diverse clinical environments, where imaging equipment and protocols differ significantly. While this could add complexity to preprocessing workflows, it would ultimately enhance the model’s applicability and usability in real-world scenarios.

Additionally, ensuring global accessibility and ease of use is crucial for the adoption of YOLOv10 in clinical settings. Open-source availability facilitates integration into workflows, while user-friendly interfaces with graphical tools can minimize the need for technical expertise. Cloud-based solutions could further enhance accessibility by eliminating hardware requirements, particularly in resource-limited settings. Providing comprehensive documentation, tutorials, and support resources would also empower practitioners to implement and utilize the model effectively. Future efforts should focus on these aspects to ensure seamless integration into diverse clinical practices.

Future research should focus on expanding the sample size and incorporating data from multiple centers with diverse populations. Such efforts would enhance the generalizability of the findings and further validate YOLOv10’s potential as a reliable tool for automated tooth detection and numbering in pediatric dentistry.

## 5. Conclusions

The results of our study demonstrate that YOLOv10 can be effectively used for tooth detection and numbering. Although it uses fewer parameters than previous versions, YOLOv10 shows comparable or even higher accuracy in performance. The application of YOLOv10 in tooth detection and numbering is expected to prevent unnecessary time loss for clinicians. We believe that this study will serve as a foundation for future research with larger sample sizes and segmentation analyses involving YOLOv10.

## Figures and Tables

**Table 1:** Precision, recall, mAP50, mAP50-95, and F1 scores by tooth number.

Tooth No.	Precision (P)	Recall (R)	mAP50	mAP50-95	F1 Score
**11**	0.964	1.000	0.995	0.779	0.982
**12**	0.995	0.897	0.977	0.673	0.943
**13**	0.908	1.000	0.993	0.783	0.951
**14**	0.966	0.958	0.992	0.733	0.962
**15**	0.962	1.000	0.995	0.731	0.980
**16**	0.839	1.000	0.986	0.840	0.913
**17**	0.979	1.000	0.995	0.822	0.989
**18**	0.793	1.000	0.978	0.752	0.885
**21**	0.967	0.933	0.990	0.736	0.950
**22**	0.993	0.933	0.990	0.744	0.962
**23**	0.959	0.967	0.993	0.767	0.963
**24**	0.967	0.974	0.994	0.720	0.971
**25**	0.966	0.947	0.987	0.687	0.956
**26**	0.829	0.967	0.980	0.856	0.893
**27**	0.907	0.978	0.988	0.790	0.941
**28**	1.000	0.938	0.995	0.726	0.968
**31**	0.936	0.970	0.993	0.760	0.953
**32**	0.977	1.000	0.995	0.781	0.988
**33**	0.994	0.900	0.984	0.812	0.944
**34**	1.000	0.938	0.995	0.769	0.968
**35**	0.965	0.921	0.989	0.800	0.943
**36**	0.880	0.975	0.991	0.889	0.925
**37**	0.786	1.000	0.968	0.804	0.880
**38**	0.967	0.900	0.908	0.652	0.932
**41**	0.932	1.000	0.983	0.729	0.965
**42**	0.986	0.933	0.986	0.722	0.959
**43**	0.871	1.000	0.989	0.785	0.931
**44**	0.960	1.000	0.993	0.816	0.980
**45**	0.898	0.967	0.989	0.779	0.931
**46**	0.916	1.000	0.974	0.854	0.956
**47**	0.947	1.000	0.972	0.808	0.973
**48**	0.929	0.900	0.905	0.580	0.914
**51**	0.656	0.800	0.712	0.337	0.721
**52**	0.681	0.715	0.835	0.502	0.697
**53**	0.907	0.926	0.948	0.635	0.916
**54**	0.914	0.972	0.992	0.692	0.942
**55**	0.963	0.988	0.959	0.719	0.975
**61**	0.816	0.898	0.962	0.400	0.855
**62**	0.911	1.000	0.995	0.588	0.953
**63**	0.922	0.912	0.969	0.628	0.917
**64**	0.946	0.921	0.985	0.714	0.933
**65**	0.805	0.868	0.908	0.668	0.835
**71**	0.660	1.000	0.995	0.0995	0.795
**72**	1.000	0.675	0.984	0.492	0.806
**73**	0.941	0.960	0.972	0.705	0.950
**74**	0.799	0.865	0.940	0.696	0.830
**75**	0.975	0.963	0.991	0.770	0.969
**81**	0.450	1.000	0.995	0.597	0.621
**82**	0.871	0.751	0.926	0.444	0.807
**83**	0.892	0.760	0.900	0.621	0.821
**84**	0.886	1.000	0.965	0.731	0.940
**85**	0.863	1.000	0.936	0.688	0.926

## Data Availability

The data presented in this study are available on request from the corresponding author due to patient confidentiality and ethical restrictions.

## Abbreviations

DLM	Deep learning model
PR	Panoramic radiograph
AI	Artificial intelligence
ML	Machine learning
YOLO	You Only Look Once
DL	Deep learning
CNN	Convolutional neural network
CLAIM	Checklist for Artificial Intelligence in Medical Imaging
STARD	Standards for the Reporting of Diagnostic Accuracy Studies
FDI	Fédération Dentaire Internationale
NMS	Non-maximum suppression
CSPNet	Cross Stage Partial Network
PAN	Path Aggregation Network
IoU	Intersection over Union
